# On homology searches by protein Blast and the characterization of the age of genes

**DOI:** 10.1186/1471-2148-7-53

**Published:** 2007-04-04

**Authors:** M Mar Albà, Jose Castresana

**Affiliations:** 1 Research Unit on Biomedical Informatics, Catalan Institution for Advanced and Research Studies, Institut Municipal d'Investigació Mèdica, Universitat Pompeu Fabra, 08003 Barcelona, Spain; 2 Department of Physiology and Molecular Biodiversity, Institut de Biologia Molecular de Barcelona, CSIC, 08034 Barcelona, Spain

## Abstract

**Background:**

It has been shown in a variety of organisms, including mammals, that genes that appeared recently in evolution, for example orphan genes, evolve faster than older genes. Low functional constraints at the time of origin of novel genes may explain these results. However, this observation has been recently attributed to an artifact caused by the inability of Blast to detect the fastest genes in different eukaryotic genomes. Distinguishing between these two possible explanations would be of great importance for any studies dealing with the taxon distribution of proteins and the origin of novel genes.

**Results:**

Here we used simulations of protein sequences to examine the capacity of Blast to detect proteins of diverse evolutionary rates in the different species of an eukaryotic phylogenetic tree that included metazoans, fungi and plants. We simulated the evolution of protein genes with the same evolutionary rates than those observed in functional mammalian genes and with among-site rate heterogeneity. Under these conditions, we found that only a very small percentage of simulated ancestral eukaryotic proteins was affected by the Blast artifact. We show that the good detectability of Blast is due to the heterogeneity of protein evolutionary rates at different sites, since only a small conserved motif in a sequence suffices to detect its homologues. Our results indicate that Blast, at least when applied within eukaryotes, only misses homologues of extremely fast-evolving sequences, which are rare in the mammalian genome, as well as sequences evolving homogeneously or pseudogenes.

**Conclusion:**

Although great care should be exercised in the recognition of remote homologues, most functional mammalian genes can be detected in eukaryotic genomes by Blast. That is, the majority of functional mammalian genes are not as fast as for not being detected in other metazoans, fungi or plants, if they had been present in these organisms. Thus, the correlation previously found between age and rate seems not to be due to a pure Blast artifact, at least for mammals. This may have important implications to understand the mechanisms by which novel genes originate.

## Background

It has been shown that novel genes, that is, genes with a restricted taxonomic distribution, evolve faster than older genes. These genes are normally identified by performing Blast searches [1] in genomes of different organisms. When genes are detected in only one species or in a group of very closely related species, these genes are called orphan genes. Orphan genes were shown to evolve very fast in bacteria [2] and *Drosophila *[3]. Genes which are not strictly orphans, but also have a narrow taxonomic distribution, such as genes present only in rodents, also showed markedly accelerated rates [4]. In addition, mammalian genes which are present only in vertebrates exhibited higher evolutionary rates than genes of precambrian origin [5]. These observations were extended to mammalian genes classified in four different ages -Old, Metazoans, Deuterostomes and Tetrapods-, which showed increasingly faster evolutionary rates in genes of more recent origin [6]. Similar conclusions could be deduced from fungi classified in different linage specificities [7]. Therefore, the relationship between age and rate seems to apply to genes of all ages and to different taxa.

The relationship between age and evolutionary rate of genes not only has importance for using age as one of many predictors for protein evolutionary rate [8,9], but also to understand the origin of novel genes. Domazet-Loso and Tautz [3] proposed a model to explain how orphan genes may arise from duplicated genes. In their scenario, one of the duplicates evolves so fast, probably by strong positive selection, that its sequence becomes unrecognizable by sequence homology searches. These genes probably acquire a new function in the lineage where they evolve. After the origination of the new sequence, evolution slows down due to the constraints imposed by the conservation of function. Albà and Castresana [6] extended this model to genes of different times of origin, and not only to orphans, as one possible explanation for the correlation found between age and rate of genes. A logical outcome from this model is that genes evolve fast in their early stages and then slow down due to increased functional constraints. This same idea has appeared several times in the literature [10-12]. Although sequence similarity is lost, the use of the protein structure might help to detect the original copy from which some of these genes arose [13,14]. Thus, according to this model, and despite talking about "novel genes", genes do not appear *de novo *in the genome, but originate from a gene duplication. The name "novel genes" would be justified because the exceptionally fast rates in their initial stages, just after the duplication, made them unrecognizable from the sequence point of view and determine their point of birth from a functional perspective. This is a simplified model that could be appropriate for the origin of most novel genes. Notable exceptions where different models may apply include the origin of slow-evolving orphan genes [3] or the formation of some novel genes from noncoding DNA [15].

In most works where Blast has been used to detect putative novel genes in other genomes, the logical concern was expressed that some of these genes may have been incorrectly classified as novel because, being fast genes, they simply were not detected by Blast in other organisms. In the case of the study of mammalian genes of different ages [6], this was supposed not to have a significant effect because the same correlation between age and rate was observed in a set of highly conserved genes, with nonsynonymous rates lower than 0.051 substitutions/position, where Blast would most probably always find homologues in all genomes. However, Elhaik et al. [16] argued that the assertion that new genes evolve faster is indeed an artifact due to the Blast relative performance in sequences of different evolutionary rates. They based their argumentation on the results from Blast searches using simulated DNA sequences of the same age and evolving at different evolutionary rates. Upon classifying these simulated sequences in different groups according to Blast detection, DNA rates had averages (but not distributions) similar to the nonsynonymous rates found in Albà and Castresana [6]. If the Blast effect could explain the results, the proposed punctuated model for the origin of orphans and other novel genes [3,6] would not be necessary, since these genes would be normal genes with just faster rates. However, there were several features in the work of Elhaik et al. [16] that made their conclusions difficult to extrapolate to other works. First, they used simulated DNA sequences and calculated DNA genetic distances, which cannot be directly compared to nonsynonymous genetic distances. Second, they performed DNA Blast searches, which are much less sensitive than protein Blast searches in which most studies are based. Third, in their simulations all positions in the sequences can experience mutations during evolution, as if they were pseudogenes, and thus it is again difficult to extrapolate these simulations to functional proteins, which typically contain motifs and are easier to detect with Blast.

An important issue regarding the power of Blast to detect homology is that the information contained in sequences degrades as these sequences accumulate mutations. However, the point at which the information is completely lost depends on the type of mutational process. In the present work, we simulated protein sequences evolving with and without among-site rate variation in the mutational process, and with evolutionary rates equivalent to those of the mammalian genome. Our results show that, when sequences were evolved with among-site rate variation, the large majority of mammalian genes were able to find their homologues in the most distant species considered in this work. This indicates that the large amount of novel genes found in the genomes of mouse and human (all of them presumably functional and therefore evolving with among-site rate variation) and the observed correlation between age and rate are not due to a pure Blast artifact. Instead, this correlation may have important biological implications, since it supports a model for the origin of novel genes that implies low constraints in the early stages after the duplication and an increase of constraints with time.

## Results and Discussion

### Amino acid rate distributions of mammalian genes

**Figure 1:**
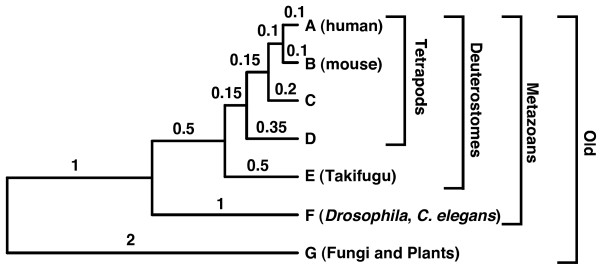
**Phylogenetic tree used in the simulations**. Numbers along the branches are proportional to the branch lengths and indicate amino acid substitutions/site. The taxonomic groupings used for the age classification of genes are also shown.

Our previous work showed that the distributions of nonsynonymous evolutionary rates of 4706 mammalian genes classified in different ages -Old, Metazoans, Deuterostomes and Tetrapods – were very different, with the youngest genes having faster rates [6]. The species used to make the age classification by means of Blast homology searches are shown in Figure 1. Since genes of different ages were of different length, and to avoid any influence from this variable, we repeated the calculations with a subset of genes coding for proteins between 300 and 500 amino acids. Due to this restriction, the total number of genes was smaller than in the original study (1558 genes distributed in 900 Old, 502 Metazoans, 134 Deuterostomes and 22 Tetrapods), but now the average length of genes was close to 400 amino acids for all ages. In addition, since our simulations were performed at the amino acid level, we calculated the protein evolutionary rate of the translated sequences (extrapolated from the human-mouse protein genetic distances) instead of the nonsynonymous rate previously calculated from the DNA sequences.

**Figure 2:**
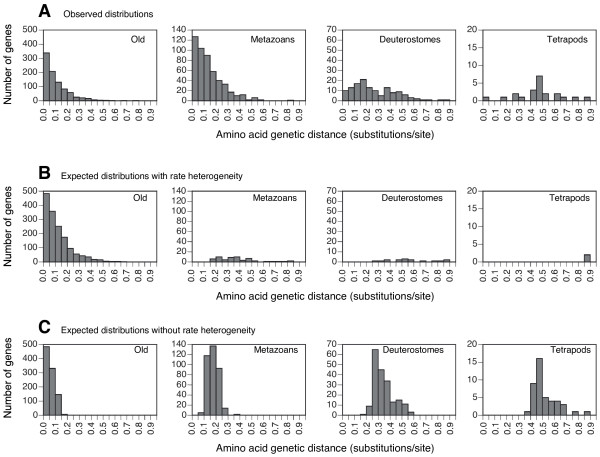
**Distributions of amino acid genetic distances in genes of different age categories**. (a) Distributions of genetic distances of the 1558 mammalian genes coding for proteins with lengths between 300 and 500 amino acids. (b) Distributions of genetic distances of the simulated genes with rate heterogeneity. (c) Distributions of genetic distances of the simulated genes without rate heterogeneity. The total number of genes for the simulations with and without rate heterogeneity were 1618 and 1578, respectively. Different pools of simulated alignments always produced very similar results (see Methods).

The shape and mean of the new rate distributions, controlling for protein length, was very similar to the distributions obtained with the original data set (Figure 2A). There was also a fourfold increase in the mean rate from the oldest to the youngest genes: 0.11 amino acid substitutions/position in Old, 0.14 in Metazoans, 0.27 in Deuterostomes, and 0.47 in Tetrapods. Genes of all age categories were involved in all types of biological processes [6], and thus it is very surprising that the oldest genes are more conserved than the younger genes. Could these rate distributions be due to a pure Blast artifact, as previously proposed [16]?

### Simulations of protein sequences

We set up our simulation experiments to know how many times the age of mammalian genes was misclassified by protein Blast. In addition, we wanted to compare the actual observations about novel genes to the results obtained with the null hypothesis of a common origin of all genes (and differential detection by Blast depending on speed of evolution). A key feature of our simulation was the use of rate heterogeneity. The rationale for using this assumption is that all mammalian sequences whose origin we wanted to elucidate are present, at least, in mouse and human (we did not use strict orphan genes, that is, genes present in a single species), and all of them have an assigned GO function. Therefore, most of these sequences are very likely to be functional and evolve under a rate heterogeneity model. The basic approach was then to simulate sequences of 400 amino acids evolving under a rate heterogeneity model, and along a given phylogenetic tree. The branch lengths of the tree were proportional to the approximate times separating the plant, animal and fungal genomes that were used to classify genes in different ages (Figure 1). Sequences C and D of this tree were included only to produce a more homogeneous taxonomic distribution and better view the simulated alignments. This tree was multiplied by different factors to obtain sequences of different evolutionary rates. We were specifically interested in knowing how Blast behaves with sequences of evolutionary rates equivalent to the rates of mammalian genes. For this purpose, we measured the exact amino acid rate of each simulation (genetic distance between A and B) and, from the performed simulations, we randomly pooled a set of genes with the same overall distribution of rates than the real ones. Then, we used Blast to classify these simulated sequences as Old, Metazoans, Deuterostomes or Tetrapods. In this setup, we know that sequences not classified as Old are misclassified due to Blast detection errors. Figure 2B shows that the number of misclassified genes is insignificant. Total numbers of misclassified genes were 57 in Metazoans, 16 in Deuterostomes, and 2 in Tetrapods (different pools always produced very similar results). If we subtracted an equivalent number of misclassified genes from the distributions of real genes (Figure 2A), these distributions would almost not change. Thus, the Blast effect seems to be very small and with no consequence for the age classification of genes. In addition, the results of these simulations do not fit the observed data, where a much higher number of novel and fast genes exists than predicted by the null hypothesis. The existence of such novel genes thus needs an explanation different from the Blast artifact.

As expected, detectability values of Blast (that is, the proportion of properly classified genes) clearly showed a decrease with faster rates. For example, most of the genes with the highest rates (around 0.825 or 0.925 substitutions/site) were clearly misclassified. But the bulk of mammalian genes is highly conserved, and for the rate categories where most of the genes are found detectability values approach 100%. This shows again that the Blast effect is not sufficiently strong as to explain the high number of novel genes found in the mammalian genome and the tendency of younger genes to evolve faster, at least when Blast is performed at the protein level and within eukaryotic genomes.

### Heterogeneity of evolution and Blast detectability

**Figure 3:**
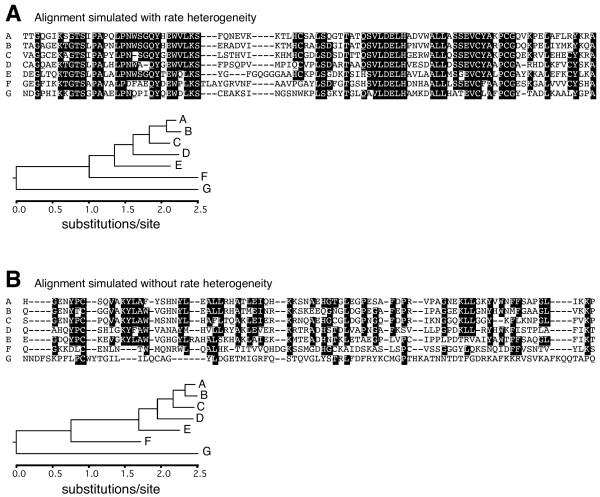
**Fragments of alignments of simulated sequences**. Simulations were done with (a) or without (b) among-site rate heterogeneity. Both simulation were performed with Rose, following the tree represented in Figure 1 multiplied by a factor of 1.5. Positions of the alignments where more than 50% of the sequences are identical are shown with black boxes. The trees recalculated from the respective complete alignments are also shown, with the scale in amino acid substitutions/site. Interestingly, despite there being a very similar genetic distance between A and G in both alignments, A finds G by Blast in the alignment evolved under rate heterogeneity (A) but not in the alignment without rate heterogeneity (B).

As explained above, an important feature of our simulations was the use of among-site rate heterogeneity. When sequences were simulated without rate heterogeneity, Blast misclassified much more sequences of the three youngest categories, affecting genes of almost all rate categories (Figure 2C). This simulation experiment is more in line with the results found by Elhaik et al. [16]. However, it is important to remark that all mammalian sequences we used have an assigned GO category, are present in at least mouse and human, and therefore are most surely functional. Functional sequences evolve in a heterogeneous manner: those sites which are most crucial for function are highly conserved, others are only moderately conserved, and others typically evolve very fast. If some conserved sites in a sequence are in close proximity, and even if the sequence has a high overall evolutionary rate, Blast will find its homologues in very distant species. As an example, figure 3A shows fragments of sequences simulated under heterogeneous evolution, which contain some conserved sequence patches, and Figure 3B shows homogeneously simulated sequences, where the conservation of all sites degrade equally with time. It is interesting to note that, despite deriving from the same tree, homogeneously evolved sequences look more divergent than heterogeneous sequences due to the lack of conserved positions in the former. On the other hand, heterogeneously evolved sequences have conserved positions, but the nonconserved positions are plagued with multiple substitutions, thus explaining that the same genetic distances are measured from both alignments. Of course, Blast behaves very differently in both types of alignments and detectability of homologues is much higher in the one evolved heterogeneously (see legend to Figure 3). Heterogeneity of evolutionary rates within a protein sequence thus explains why Blast sensitivity is quite decoupled from overall divergence, at least for moderately divergent sequences.

### Trivial correlation between rate and age in simulated sequences

Simulated genes that escape Blast detection, and are thus not classified as Old, will tend to be fast evolving genes, creating an obvious correlation, as can be seen in Figure 2B, and even more exaggeratedly in Figure 2C. However, this cannot explain the observed relationship between gene age and evolutionary rate [16], since the fact that genes that escape Blast detection evolve fast does not mean that all fast genes escaped Blast detection. As indicated above, what it is necessary is to subtract the misclassified genes from the set of real genes, and analyze if the correlation is maintained in the real genes. We have shown that the proportion of misclassified genes is so small – when sequences are simulated heterogeneously and with rates like those observed in mammalian genes – that the relationship existing between age and rate in the real data set cannot be explained only by a Blast artifact.

## Conclusion

The appearance of novel genes in genomes is one of the most intriguing aspects in evolutionary genomics. The danger of miss-classifying fast-evolving genes as novel by Blast has been clearly exposed by Elhaik et al. for homogeneously evolved sequences [16]. We have shown that, specifically for mammalian functional genes and for their detectability by protein Blast within higher eukaryotes, the loss of sensitivity for the fastest genes does not affect significantly the overall assignment of the age and rate of genes. Therefore, these data support a more complex scenario for functional sequences that involves the existence of a large number of novel genes, many of which happen to be relatively fast evolving. This correlation between age and rate may have a crucial biological interest, because it is consistent with a model of origin of novel genes that assumes that these genes arose through a gene duplication and an initial phase of exceptionally high evolutionary rates. The end of this phase, and the beginning of negative selection, would indicate the point of birth of these genes. From here on, increased functional constraints would be reflected in lower evolutionary rates. However, the history of every gene is very complex and may include many particularities that cannot be covered by the general type of study presented here. More detailed studies will be necessary to see if this model of origin of novel genes is true and to understand the molecular evolution of every particular novel gene.

## Methods

### Gene dataset

The results of Blast are highly dependent on the length of the gene used as query. Since mammalian genes of different ages were of different length [6], and to avoid any inference from this factor, we extracted the genes encoding proteins between 300 and 500 amino acids from the dataset in [6]. Now the number of genes was smaller (1558), but the average length of genes was close to 400 amino acids for all ages (with no statistical differences in length among them). The distribution of amino acid substitution rates of genes from this subset was very similar to the one in the original, larger dataset. The list of genes used is given in additional file 1.

### Protein sequence evolution simulations

We simulated protein sequences of 400 amino acids by means of Rose [17]. This program allows the simulation of different substitution rates in different positions with a predetermined spatial pattern. We extracted spatial patterns of rate heterogeneity from 14 different eukaryotic protein alignments using TreePuzzle [18] with a model of rate heterogeneity that assumed Gamma distributed rates with 16 rate categories. In particular, we took, for each alignment position, the category and associated relative rate that contributed the most to the likelihood. These relative rates were then used by Rose to simulate different positions with different rates. The 14 alignments used to extract relative rates contained a similar set of species than that of the eukaryotic genomes used in Albà and Castresana [6]. Most heterogeneity patterns produced similar results in terms of detectability by Blast. The thresholds for gap insertion and for gap deletion in the simulations by Rose were set to 0.0001. The PAM model of amino acid substitution was used. Using these parameters, random ancestral sequences were evolved along a phylogenetic tree with branch lengths proportional to the approximate times separating the genomes used in Albà and Castresana [6] (Figure 1). Sequences C and D of this tree were included only to better view the alignments. This tree was multiplied by 15 different factors (from 0.025 to 5.5) to obtain sequences of different genetic distances, that is, evolving at different evolutionary rates. One thousand simulations were performed for every rate heterogeneity pattern (derived from each of the 14 alignments) and evolutionary rate, making a total of 210,000 simulations (14 heterogeneity patterns × 15 evolutionary rates × 1000 simulations). In addition, we also simulated another set of 210,000 alignments without using rate heterogeneity but with all other parameters in the Rose program being identical.

### Blast searches and age classification

The equivalent to the human sequence in each simulation (sequence A) was used to search by protein Blast the database of all other simulated sequences. The E-value used as threshold was 10^-8 ^(10^4 ^smaller than in Albà and Castresana [6] to account for databases approximately 10^4 ^smaller in the searches performed in the present work). Genes were classified according to the Blast hits. Sequences present in B, E, F and G were classified as "Old", present in B, E, and F but not in G as "Metazoans", present in B and E but not in F and G as "Deuterostomes", and present only in B as "Tetrapods".

Protdist of the Phylip package [19] with the JTT model of evolution [20] was used to estimate the protein genetic distance between human and mouse sequences of the real data set and between the simulated A and B sequences (equivalent of human and mouse). These genetic distances were then used as surrogates of evolutionary rate. The rate measured with Protdist in the simulations is normally slightly smaller than the initial rate used for the simulation, particularly for the highest rates, due to the saturation of the measured genetic distances, but it provides a better comparison with the rate measured by the same method in the real genes. To compare the distributions of rates (see below) both the real and simulated alignments were assigned an evolutionary rate category according to 0.025 substitutions/position intervals.

From each set of 210,000 simulated alignments we selected pools of genes belonging to different rate categories in such a way as to maintain, in each pool, the same evolutionary rate distribution than that observed in the 1558 real mammalian genes. This was achieved by using a probability of selection of each simulation proportional to the frequency of its evolutionary rate category in the real dataset divided by the frequency of its category in the simulated dataset. Each pool contained a number of simulations close to 1558, the number of genes in the real dataset, and all extracted pools produced essentially the same results. This strategy made unnecessary the calculation of explicit Blast detectability values for each rate category and made easier the comparison of expected and observed results. As all genes were evolved along the same tree, as if they were Old, the number of real genes expected to escape Blast detection is directly reflected in the distributions of simulated genes falling in the "Metazoans", "Deuterostomes", and "Tetrapods" categories.

## Authors' contributions

M.M.A and J.C. conceived the study, performed the experiments and wrote the manuscript. Both authors read and approved the final manuscript.

## Supplementary Material

Additional file 1 List of genes used in the study. The ENSEMBL Gene ID for the human and mouse genes, and the estimated age category of each gene is given. Click here for file
